# Effects of Electrical Remodeling on Atrial Fibrillation in Diabetes Mellitus

**DOI:** 10.31083/j.rcm2401003

**Published:** 2023-01-03

**Authors:** Ling-ling Qian, Xiao-yu Liu, Xiao-yan Li, Fan Yang, Ru-Xing Wang

**Affiliations:** ^1^Department of Cardiology, Wuxi People’s Hospital Affiliated to Nanjing Medical University, 214023 Wuxi, Jiangsu, China

**Keywords:** atrial fibrillation, diabetes mellitus, electrical remodeling, sodium channels, calcium channels, potassium channels, late sodium channels

## Abstract

Atrial fibrillation (AF) is one of the most common arrhythmias in medical practice. Diabetes mellitus (DM) is one of the independent risk factors for atrial fibrillation. The increased morbility of atrial fibrillation in diabetes mellitus is related to both structural and electrical remodeling of atrium. Based on studies of atrial electrophysiological changes in diabetes mellitus, this article focuses on the electrical remodeling of atrial cardiomyocytes, including remodeling of sodium channels, calcium channels, potassium channels and other channels, to provide the basis for the clinical management of antiarrhythmic drugs in diabetic patients with atrial fibrillation.

## 1. Introduction

Atrial fibrillation (AF) is one of the most common arrhythmias in medical practice worldwide [1]. Traditionally, AF can be classified into five patterns: first diagnosed, paroxysmal, persistent, long-standing persistent and permanent AF [2]. AF patients have an increased risk of congestive heart failure and stroke, resulting in severe disability and death [3]. Diabetes mellitus (DM) is one of major hazard factors for AF [4, 5, 6]. Subclinical AF episodes occur frequently in type 2 diabetes mellitus (T2DM) patients and are associated with increased thromboembolic risk [7]. Moreover, treatment of AF appears to be more challenging in patients with DM. Outcomes of AF ablation are worse in patients with DM compared to the general population and arrhythmia recurrence is significantly higher in the DM group compared to the non-DM group [8, 9]. Both AF and DM are currently prominent global public health issues [10]. However, the underlying mechanisms of AF in DM have not been completely investigated.

DM predisposes to AF due to several factors, such as atrial remodeling, autonomic system dysfunction [11] and epigenetic regulation [12]. Atrial remodeling includes structural remodeling and electrical remodeling. Atrial structural remodeling was found in both type 1 and type 2 DM animal models [5, 13]. There are evidences demonstrating that DM is associated with disordered arrangement and higher cross-sectional areas of atrial cardiomyocytes, as well as increased interstitial fibrosis and inflammation [5]. Ultrastructural studies of DM cardiomyocytes also showed irregularly arranged myofibrils, degenerated Z-lines, and swollen, vacuolated mitochondria with fragmentation [14]. In addition to structural remodeling, investigators have studied the ionic mechanisms that underlie the electrical remodeling of AF in DM [15]. This article reviews the remodeling of ion channels in atrial myocytes with DM and their related mechanisms, so as to provide the basis for the clinical treatment of antiarrhythmic drugs used in patients with diabetic AF.

## 2. AF Incidence and Alterations of Atrial Electrophysiological Characteristics in Diabetes

In previous studies, diabetic animals have shown a high susceptibility for induced AF, with a significantly higher incidence of AF and a longer AF duration after atrial burst stimulation [4, 5, 6, 16, 17, 18, 19, 20, 21, 22]. The electrocardiogram and electrophysiological parameters of diabetic animals have been reported in humans [23] and in different animal models [24]. Diabetic animals often show irregularities in atrial depolarization as P-wave prolongation and increased P-wave dispersion, leading to impulse generation or conduction abnormalities [24]. Lower heart rate (HR), prolonged rhythm-to-rhythm (RR) interval and similar Q wave-R wave-S wave (QRS) duration, onset of wave Q to the end of wave T (QT interval), QTc interval (corrected QTc interval) were observed in type 1 diabetic Sprague Dawley (SD) rats induced by streptozotocin (STZ) (6 weeks after treatment) [4]. No significant differences were found in sinus cardiac length (SCL), left atrial (LA) effective refractory period (LA-ERP), right atrial (RA) ERP (RA-ERP), inter atrial conduction time (IACT), RA-ventricular conduction time (RA-VCT) and LA-ventricular conduction time (LA-VCT) between control and diabetic groups. In contrast, the conduction velocity of atria was slower and conduction in homogeneity was notably increased in diabetic rats with a higher incidence of AF. In STZ-induced diabetic Wistar rats (8 weeks after treatment), IACT was longer, LA-ERP and RA-ERP were shorter than control rats [5]. However, no significant differences were observed in atrioventricular Wenckebach cycle length (AV-WCL) and HR. The incidence of AF was also increased in a type 2 DM (T2DM) animal model [16]. In high fat diet (HFD) and low dose STZ treated SD male rats, LA conduction velocity was significantly lower as shown by mapping images. The IACT was longer and SCL, AV-WCL, RA-ERP and LA-ERP were not statistically different [17]. In 20-week-old Zucker Diabetic Fatty (ZDF) rats, SCL, the time of sinus node recovery and corrected sinus node recovery were noteworthy longer than those in the control group [18]. The electrocardiogram (ECG) of T2DM model Goto-Kakizaki (GK) rats showed accelerated HR, irregular P waves, separation of QRS and P waves, and partial blockade of electrical conduction [25]. Atrial effective refractory period (AERP), duration of P-wave, and the time from the onset of the P wave until the R wave (PR interval) and RR interval were longer in db/db type 2 diabetic mice (16 and 20 weeks of age) [13]. In summary, abnormal electrophysiological parameters were observed in both type 1 and type 2 DM models, including chemically induced type 1 DM, metabolic type 2 DM with mild to medium pancreatic injury followed by diet induced insulin resistance , and genetically hyperglycemic animals (Table 1, Ref. [4, 5, 13, 16, 17, 18, 25]).

**Table 1.** **Overview of atrial alterations in electrophysiological parameters of diabetes mellitus**.

Diabetes mellitus type	Animal model	Atrial alterations in electrophysiological parameters	References
Chemically induced T1DM	STZ-induced diabetic SD rats (6 weeks after treatment)	HR↓, RR interval↑, conduction velocity↓, conduction inhomogeneity↑	[4]
	STZ-induced diabetic Wistar rats (8 weeks after treatment)	IACT↑, LA-ERP↓, RA-ERP↓	[5]
Metabolic T2DM	HFD and low-dose STZ treatment SD rats	LA-CV↓, IA-CT↑	[16]
	HFD and low-dose STZ treatment SD rats	IACT↑	[17]
Genetically T2DM	20-week-old ZDF rats	SCL↑, sinus node recovery time↑ and corrected sinus node recovery time↑	[18]
	Goto-Kakizaki rats	HR↑, irregular P waves, separation of P and QRS waves	[25]
	db/db diabetic mice (16 and 20 weeks old)	AERP↑, duration of P-wave↑, interval of PR↑, and interval of RR↑	[13]

T1DM, type 1 diabetes mellitus; T2DM, type 2 diabetes mellitus; STZ, streptozotocin; SD, Sprague Dawley; HFD, high fat diet; HR, heart rate; IACT, inter atrial conduction time; LA-ERP, left atrial-effective refractory period; RA-ERP, right atrial-effective refractory period; LA-CV, left atrial conduction velocity; SCL, sinus cardiac length; AERP, atrial effective refractory period.

## 3. Ion Channel Remodeling of Atrial Myocytes in Diabetes

The abnormal electrophysiological parameters in diabetes are closely related to the changes of action potentials (AP) in atrial myocytes. AP duration (APD) occurs in diabetic animals with higher ${\mathrm{APD}}_{50}$ and ${\mathrm{APD}}_{90}$ [18, 25]. Prolonged APDs result from up- or down-expression and activation or inactivation of different kinds of ion channels that form depolarizing and repolarizing currents, such as sodium channels, calcium channels, potassium channels, late sodium channels and other channels.

### 3.1 Sodium Channels

The voltage-gated sodium channel current ($I_{\text{Na}}$) is widely found in the atrium and is one of the most important depolarization cation channels in the cardiomyocyte membrane [26]. It is the major determinant of the upstroke of AP. Proteins of ${\mathrm{Na}}_{\text{V}}$1.5 (encoded by *SCN5A* gene) are responsible for $I_{\text{Na}}$ [27]. In Akita type 1 diabetic mice, $I_{\text{Na}}$ was measured both in isolated RA and LA myocytes [21]. Current-voltage (I-V) curves of atrial myocytes demonstrated that $I_{\text{Na}}$ was reduced. The decreased $I_{\text{Na}}$ density of diabetic mice occurred in association with a decline of maximum conductance (Gmax) and a mode straight shift of the voltage dependence of activation. Voltage dependence of inactivation was not altered in atrial myocytes of Akita mice. The expression of *SCN5A* mRNA and ${\mathrm{Na}}_{\text{V}}$1.5 proteins were reduced in the atrium of Akita mice compared with normal controls. The alterations of $I_{\text{Na}}$ resulted in prolonged P-wave duration, and reduced atrial conduction velocity in Akita mice. Acute insulin treatment increased $I_{\text{Na}}$ due to enhanced insulin signaling through activation of phosphatidylinositol 3,4,5-triphosphate (PIP3).

In atrial myocytes of db/db mice [13], $I_{\text{Na}}$ amplitude, $I_{\text{Na}}$ steady-state activation, or fast and slow time constants of $I_{\text{Na}}$ activation were similar to control mice. The steady-state inactivation curve was shifted to the right, which suggested a larger window current. *SCN5A* mRNA and ${\mathrm{Na}}_{\text{V}}$1.5 protein levels was similar in db/db atrium compared with control. In metabolic type 2 DM with STZ-injection followed by diet induced insulin resistance, the density of $I_{\text{Na}}$ were similar in the control and T2DM rat myocytes [24].

Sodium channels are activated during the depolarization phase and then rapidly deactivated. However, some channels reopen as a late or persistent sodium current ($I_{\text{Na-L}}$) that participate in repolarization [28]. The basal $I_{\text{Na-L}}$ is mainly generated from the ${\mathrm{Na}}_{\text{V}}$1.5 isoform and is regulated by calmodulin-dependent kinase II [29]. Several studies show that the increase of $I_{\text{Na-L}}$ can markely prolong the duration of AP in cardiomyocytes and is important in the development of AF [30, 31, 32]. $I_{\text{Na-L}}$ was increased in isolated atrial myocytes of DM mice compared to controls [33]. In diabetic mice, the application of the $I_{\text{Na-L}}$ inhibitor (GS967) inhibited $I_{\text{Na-L}}$, shortened APD, and reduced the incidence of AF by high-frequency electrical stimuli. A recent study in knock-in mice fed a high fat diet, which ablates phosphorylation of the ${\mathrm{Na}}_{\text{V}}$1.5 channel and prevents augmentation of $I_{\text{Na-L}}$, increased AF inducibility [31]. In conclusion, the increased susceptibility to AF in diabetic mice was associated with increased $I_{\text{Na-L}}$ and the subsequent prolongation of AP.

### 3.2 Calcium Channels

The voltage gated calcium channel is another important cation influx channel. The L-type calcium current ($I_{\text{CaL}}$) contributes to a depolarizing current which is actived during the repolarization phase of AP. It is responsible for the maintenance of the platform stage. Proteins of ${\mathrm{Ca}}_{\text{V}}$1.1~1.4 and ${\mathrm{Ca}}_{\text{V}}$3.1~3.3 are responsible for $I_{\text{CaL}}$ and T-type ${\mathrm{Ca}}^{2+}$ currents, respectively.

In atrial myocytes of STZ-induced diabetes, the maximum current density of $I_{\text{CaL}}$ was significantly higher compared with control. The steady-state $I_{\text{CaL}}$ activation curve was shifted to the left and the activation slope factor was decreased, while the inactivation curve was shifted to the right and the inactivation slope factor was higher in the diabetic group [6]. These results suggested the $I_{\text{CaL}}$ was easily activated and was difficult to be inactivated in DM. ${\mathrm{Ca}}_{\text{V}}$1.2 protein expression was also increased in the diabetic atrium. Selective inhibition of protein kinase C (PKC)-β using ruboxistaurin (RBX) can reduce nuclear factor kappa-B (NF-κB)/transforming growth factor-β (TGF-β)/${\mathrm{Ca}}_{\text{V}}$1.2 expression and $I_{\text{CaL}}$ activation, and inhibit abnormal atrial remodeling in diabetic rats. In ZDF rats, the protein expression level of ${\mathrm{Ca}}_{\text{V}}$1.2 in the atrium and current density of $I_{\text{CaL}}$ were significantly lower in the atrial myocytes, while the kinetics of $I_{\text{CaL}}$ were similar to the control group [18]. In the atrium of metabolic type2 diabetic rats, ${\mathrm{Ca}}_{\text{V}}$1.2 mRNA and protein expression were significantly decreased, whereas the level of ${\mathrm{Ca}}_{\text{V}}$3.1 was upregulated [14]. $I_{\text{CaL}}$ was reduced and the T-type ${\mathrm{Ca}}^{2+}$ current was increased in diabetic atrial myocytes. Long term rosuvastatin treatment alleviated these pathological changes in diabetic rats. The results of studies involving $I_{\text{CaL}}$ have not been consistent, and may be related to the use of different animal models and the duration of diabetes.

### 3.3 Potassium Channels

There are several types of potassium channels in cardiomyocytes. It has been reported that the main repolarizing potassium currents ($I_{\text{K}}$) are transient outward potassium currents ($I_{\text{to}}$), rapid-delayed rectifier potassium currents ($I_{\text{Kr}}$), slow-delayed rectifier potassium currents ($I_{\text{Ks}}$) and steady-state potassium currents ($I_{\text{ss}}$) in the human heart ventricle, while they are fast transient-outward potassium currents ($I_{\text{to, f}}$), ultra-rapid delayed rectifier potassium current ($I_{\text{Kur}}$) and $I_{\text{Ks}}$ currents in the atrium. $I_{\text{to}}$ and $I_{\text{Kur}}$ participate in the phase 1 repolarization process of myocardial AP, and $I_{\text{Kr}}$, $I_{\text{Ks}}$ participate in the phase 2 and phase 3 repolarization process of AP. Proteins of $K_{\text{V}}$4.2 (encoded by *KCND2*) and $K_{\text{V}}$4.3 (encoded by *KCND3*) are responsible for $I_{\text{to}}$, and $K_{\text{V}}$1.5 proteins (encoded by *KCNA5*) are responsible for $I_{\text{Kur}}$ [34].

Bohne *et al*. [13] found atrial $I_{\text{K}}$, mainly including $I_{\text{to}}$ and the $I_{\text{Kur}}$, were decreased in atrial myocytes of db/db mice. The decrease of $I_{\text{to}}$ occurred in association with reductions in the expression of *KCND2* mRNA and $K_{\text{V}}$4.2 proteins (mRNAs for *KCND3* were reduced and $K_{\text{V}}$4.3 proteins were similar). The reduction in $I_{\text{Kur}}$ was not related to mRNA or protein expression (no differences in *KCNA5* mRNA or $K_{\text{V}}$1.5 protein levels). There were no differences in calcium-activated potassium currents in atrial myocytes of db/db mice. Atrial current density of $I_{\text{to}}$ and $I_{\text{Kur}}$ in ZDF diabetic rats was less than that in controls and the expression levels of the protein $K_{\text{V}}$4.3 and $K_{\text{V}}$1.5 were significantly downregulated [18]. No significant differences were found in the kinetics of $I_{\text{to}}$. Polina *et al*. [21] also found $I_{\text{K}}$ carried by $K_{\text{V}}$1.5 channels were reduced in type 1 diabetic Akita mice. They measured $I_{\text{K}}$ in atrial myocytes with and without a prepulse to inactivate $I_{\text{to}}$. Peak total $I_{\text{K}}$ was reduced in diabetic atrial myocytes while $I_{\text{to}}$ (the difference currents between measurements with and without the inactivating prepulse) were similar between wildtype and diabetic mice. The $I_{\text{Kur}}$, as measured by 4-aminopyridine sensitive $I_{\text{K}}$, was reduced, and western blot showed no differences in $K_{\text{V}}$4.2 and $K_{\text{V}}$4.3 protein levels of the atrium from wild-type and diabetic mice; however $K_{\text{V}}$1.5 protein was reduced with no difference in mRNA expression. Inward rectifier $K^{+}$ currents ($I_{\text{k1}}$) mainly affected resting membrane potential. No significant difference in $I_{\text{k1}}$ densities were found between control and diabetic atrial myocytes [13, 24].

There are numerous studies showing that small conductance calcium-activated potassium channels (SK channels) play important roles in diabetic AF. The SK channels have three isoforms including SK1 ($K_{\text{Ca}}$2.1, encoded by *KCNN1*), SK2 ($K_{\text{Ca}}$2.2, encoded by *KCNN2*) and SK3 ($K_{\text{Ca}}$2.3, encoded by *KCNN3*). The SK currents were significantly reduced and the AP duration was prolonged in atrial myocytes of GK rats [25]. Compared with control rats, the expression of SK2 channel was decreased and the expression of the SK3 channel was increased in atrial myocytes of GK rats. Metformin reversed SK channel alterations in the diabetic atrium. Liu *et al*. [35] also reported that SK2 protein levels was decreased and SK3 protein elevels were increased in the atrium of T2DM rats. Metformin treatment prevents the SK channel alterations by inhibiting the PKC/extracellular signal regulated kinase pathway. Long term treatment of metformin also upregulated the SK2 channel and downregulated the SK3 channel by inhibiting the nicotinamide adeninedinucleotide phosphate oxidase 4/p38 mitogen-activated protein kinase (MAPK) signaling pathway [36].

### 3.4 Other Channels

Howarth *et al*. [37] evaluated gene expression in the sinoatrial node of GK rats and found hyperpolarization-activated cyclic nucleotide-gated channels (HCN) were downregulated. The reduction of HCN isoforms were also reduced in the sinoatrial node of diabetic rats induced with STZ injection, indicating HCN might be an important contributor to the dysfunction of sinoatrial node in DM [38]. mRNA and protein expressions of hyperpolarization-activated cyclic nucleotide-gated channel 2 (HCN2) were reduced exclusively in the ventricles of STZ rats [39]. However, HCN2 expression in the atrium of STZ rats and H9c2 cells treated with high glucose were unchanged.

Higher protein expression levels of ${\mathrm{Na}}^{+}$-${\mathrm{Ca}}^{2+}$ exchanger current (NCX) were observed in the STZ-induced diabetic group [6]. Yang *et al*. [4] observed the electrophysiological abnormalities of diabetic rats were accompanied by more severe oxidative stress and higher protein expression of NCX in the atrium. The protein level of NCX in the atrial tissue of diabetic rats was upregulated without alterations in mRNA. Allopurinol (a xanthine oxidase inhibitor) intervention can downregulate its protein level, which indicates that NCX activation plays a key role in diabetic electrical remodeling of the atrium, and antioxidant treatment improves electrical remodeling by inhibiting NCX expression.

## 4. Conclusions

**Fig. 1.**
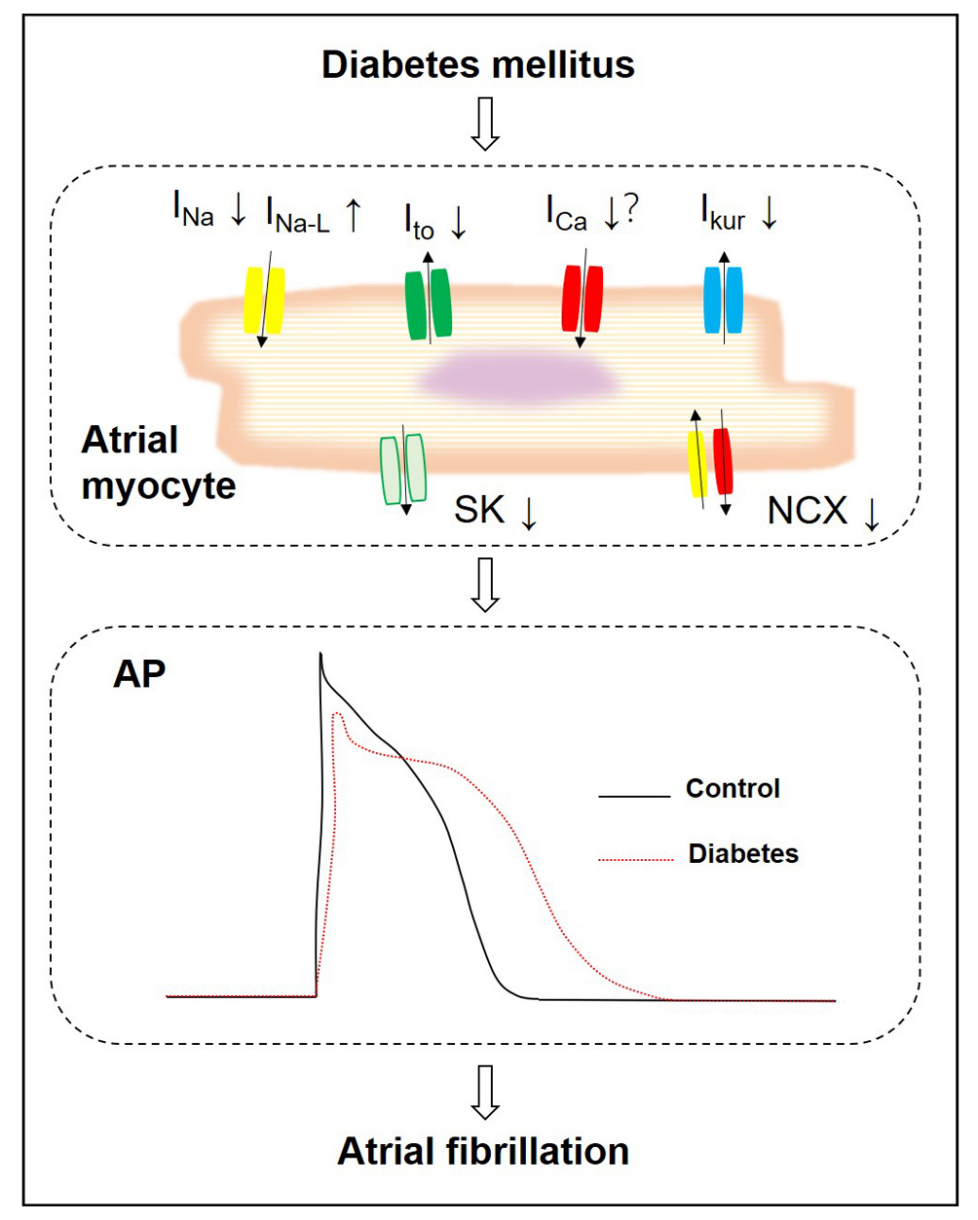
**Overview of ion channels remodeling contributing to the action potential alteration of the atrial myocytes in diabetes mellitus**. In diabetes mellitus, $I_{\text{Na}}$, $I_{\text{Na-L}}$, $I_{\text{Ca}}$, $I_{\text{to}}$, $I_{\text{Kur}}$, SK, NCX in atrium myocytes are altered, resulting in prolonged action potential duration and reduced atrial conduction velocity, increased incidence of atrial fibrillation. $I_{\text{Na}}$, the voltage-gated sodium channel current; $I_{\text{Na-L}}$, the late sodium current; $I_{\text{Ca}}$, the voltage gated calcium current; $I_{\text{to}}$, the transient outward potassium currents; $I_{\text{Kur}}$, the ultra-rapid delayed rectifier potassium current; SK, that small conductance calcium-activated potassium channels; NCX, the ${\mathrm{Na}}^{+}$-${\mathrm{Ca}}^{2+}$ exchanger current.

AF contributes to increase morbidity and mortality, especially in the DM population. Rhythm control is important to treat AF [40] and catheter ablation is the most effective treatment for AF [2]. However, success rate of ablation in diabetic patients remains lower compared to the general population particularly for those with persistent AF [8]. This is likely due to the complex substrate of AF in patients with diabetes, which may be related to chronic inflammation [41], sarcoplasmic endoplasmic reticulum calcium ATPase (SERCA) levels [42] or epigenetics, such as altered expression of microRNA [12] in AF patients. Anti-inflammatory agents may reduce AF recurrence post ablation [41]. Selective microRNA therapy, by upregulation or downregulation by microRNA, may be used to treat AF to prevent cardiac structural and electrical remodeling [12]. Various remodeling of ion channels occurs in diabetes, including the sodium channels, calcium channels, potassium channels and others, resulting in abnormal electrophysiological parameters of the atrium and increases the incidence of AF (Fig. 1). However, how these ion channels are regulated in the diabetic atrium is not fully understood. Therefore, molecular mechanisms of atrial electrical remodeling in diabetes need to be further explored, which may provide new targets for prevention and treatment of AF in diabetes mellitus.

## References

[1] Zhong W, Yang H, Wang Y, Yang Y, Guo C, Wang C (2021). Proteomic profiles of patients with atrial fibrillation provide candidate biomarkers for diagnosis. *International Journal of Cardiology*.

[2] Hindricks G, Potpara T, Dagres N, Arbelo E, Bax JJ, Blomström-Lundqvist C (2021). 2020 ESC Guidelines for the diagnosis and management of atrial fibrillation developed in collaboration with the European Association for Cardio-Thoracic Surgery (EACTS): The Task Force for the diagnosis and management of atrial fibrillation of the European Society of Cardiology (ESC) Developed with the special contribution of the European Heart Rhythm Association (EHRA) of the ESC. *European Heart Journal*.

[3] Peng X, Li L, Zhang M, Zhao Q, Wu K, Bai R (2020). Sodium-Glucose Cotransporter 2 Inhibitors Potentially Prevent Atrial Fibrillation by Ameliorating Ion Handling and Mitochondrial Dysfunction. *Frontiers in Physiology*.

[4] Yang Y, He J, Yuan M, Tse G, Zhang K, Ma Z (2020). Xanthine oxidase inhibitor allopurinol improves atrial electrical remodeling in diabetic rats by inhibiting CaMKII/NCX signaling. *Life Sciences*.

[5] Xue X, Ling X, Xi W, Wang P, Sun J, Yang Q (2020). Exogenous hydrogen sulfide reduces atrial remodeling and atrial fibrillation induced by diabetes mellitus via activation of the PI3K/Akt/eNOS pathway. *Molecular Medicine Reports*.

[6] Wang H, Xu Y, Xu A, Wang X, Cheng L, Lee S (2020). PKCbeta/NF-kappaB pathway in diabetic atrial remodeling. *Journal of Physiology and Biochemistry*.

[7] Marfella R, Sasso FC, Siniscalchi M, Cirillo M, Paolisso P, Sardu C (2013). Brief Episodes of Silent Atrial Fibrillation Predict Clinical Vascular Brain Disease in Type 2 Diabetic Patients. *Journal of the American College of Cardiology*.

[8] Creta A, Providência R, Adragão P, de Asmundis C, Chun J, Chierchia G (2020). Impact of Type-2 Diabetes Mellitus on the Outcomes of Catheter Ablation of Atrial Fibrillation (European Observational Multicentre Study). *The American Journal of Cardiology*.

[9] Wang A, Truong T, Black-Maier E, Green C, Campbell KB, Barnett AS (2020). Catheter ablation of atrial fibrillation in patients with diabetes mellitus. *Heart Rhythm O2*.

[10] Choi YJ, Han KD, Choi EK, Jung JH, Lee SR, Oh S (2021). Alcohol Abstinence and the Risk of Atrial Fibrillation in Patients With Newly Diagnosed Type 2 Diabetes Mellitus: A Nationwide Population-Based Study. *Diabetes Care*.

[11] Rizzo MR, Sasso FC, Marfella R, Siniscalchi M, Paolisso P, Carbonara O (2015). Autonomic dysfunction is associated with brief episodes of atrial fibrillation in type 2 diabetes. *Journal of Diabetes and its Complications*.

[12] Sardu C, SANtamaria M, Paolisso G, Marfella R (2015). MicroRNA expression changes after atrial fibrillation catheter ablation. *Pharmacogenomics*.

[13] Bohne LJ, Jansen HJ, Daniel I, Dorey TW, Moghtadaei M, Belke DD (2021). Electrical and structural remodeling contribute to atrial fibrillation in type 2 diabetic db/db mice. *Heart Rhythm*.

[14] Pan Y, Li B, Wang J, Li X (2016). Rosuvastatin Alleviates Type 2 Diabetic Atrial Structural and Calcium Channel Remodeling. *Journal of Cardiovascular Pharmacology*.

[15] Bohne LJ, Johnson D, Rose RA, Wilton SB, Gillis AM (2019). The Association Between Diabetes Mellitus and Atrial Fibrillation: Clinical and Mechanistic Insights. *Frontiers in Physiology*.

[16] Gong M, Yuan M, Meng L, Zhang Z, Tse G, Zhao Y (2020). Wenxin Keli Regulates Mitochondrial Oxidative Stress and Homeostasis and Improves Atrial Remodeling in Diabetic Rats. *Oxidative Medicine and Cellular Longevity*.

[17] Shao Q, Meng L, Lee S, Tse G, Gong M, Zhang Z (2019). Empagliflozin, a sodium glucose co-transporter-2 inhibitor, alleviates atrial remodeling and improves mitochondrial function in high-fat diet/streptozotocin-induced diabetic rats. *Cardiovascular Diabetology*.

[18] Fu L, Rao F, Lian F, Yang H, Kuang S, Wu S (2019). Mechanism of electrical remodeling of atrial myocytes and its influence on susceptibility to atrial fibrillation in diabetic rats. *Life Sciences*.

[19] Linz D, Hohl M, Dhein S, Ruf S, Reil J, Kabiri M (2016). Cathepsin a mediates susceptibility to atrial tachyarrhythmia and impairment of atrial emptying function in Zucker diabetic fatty rats. *Cardiovascular Research*.

[20] Uemura K, Kondo H, Ishii Y, Kobukata M, Haraguchi M, Imamura T (2016). Mast Cells Play an Important Role in the Pathogenesis of Hyperglycemia-Induced Atrial Fibrillation. *Journal of Cardiovascular Electrophysiology*.

[21] Polina I, Jansen HJ, Li T, Moghtadaei M, Bohne LJ, Liu Y (2020). Loss of insulin signaling may contribute to atrial fibrillation and atrial electrical remodeling in type 1 diabetes. *Proceedings of the National Academy of Sciences of the United States of America*.

[22] Maria Z, Campolo AR, Scherlag BJ, Ritchey JW, Lacombe VA (2020). Insulin Treatment Reduces Susceptibility to Atrial Fibrillation in Type 1 Diabetic Mice. *Frontiers in Cardiovascular Medicine*.

[23] Inanir M, Gunes Y, Sincer I, Erdal E (2020). Evaluation of Electrocardiographic Ventricular Depolarization and Repolarization Variables in Type 1 Diabetes Mellitus. *Arquivos Brasileiros de Cardiologia*.

[24] Li B, Pan Y, Li X (2016). Type 2 Diabetes Induces Prolonged P-wave Duration without Left Atrial Enlargement. *Journal of Korean Medical Science*.

[25] Fu X, Pan Y, Cao Q, Li B, Wang S, Du H (2018). Metformin restores electrophysiology of small conductance calcium-activated potassium channels in the atrium of GK diabetic rats. *BMC Cardiovascular Disorders*.

[26] Kaufmann SG, Westenbroek RE, Maass AH, Lange V, Renner A, Wischmeyer E (2013). Distribution and function of sodium channel subtypes in human atrial myocardium. *Journal of Molecular and Cellular Cardiology*.

[27] Veerman CC, Wilde AAM, Lodder EM (2015). The cardiac sodium channel gene SCN5a and its gene product NaV1.5: Role in physiology and pathophysiology. *Gene*.

[28] Zaza A, Rocchetti M (2013). The late Na+ current–origin and pathophysiological relevance. *Cardiovascular Drugs and Therapy*.

[29] Song Y, Belardinelli L (2017). Basal late sodium current is a significant contributor to the duration of action potential of guinea pig ventricular myocytes. *Physiological Reports*.

[30] Song Y, Belardinelli L (2017). Enhanced basal late sodium current appears to underlie the age-related prolongation of action potential duration in guinea pig ventricular myocytes. *Journal of Applied Physiology*.

[31] Song Y, Shryock JC, Belardinelli L (2008). An increase of late sodium current induces delayed afterdepolarizations and sustained triggered activity in atrial myocytes. *American Journal of Physiology-Heart and Circulatory Physiology*.

[32] Poulet C, Wettwer E, Grunnet M, Jespersen T, Fabritz L, Matschke K (2015). Late Sodium Current in Human Atrial Cardiomyocytes from Patients in Sinus Rhythm and Atrial Fibrillation. *PLoS ONE*.

[33] Jin X, Jiang Y, Xue G, Yuan Y, Zhu H, Zhan L (2019). Increase of late sodium current contributes to enhanced susceptibility to atrial fibrillation in diabetic mice. *European Journal of Pharmacology*.

[34] Qian L, Sun X, Yang J, Wang X, Ackerman MJ, Wang R (2021). Changes in ion channel expression and function associated with cardiac arrhythmogenic remodeling by Sorbs2. *Biochimica et Biophysica Acta - Molecular Basis of Disease*.

[35] Liu C, Hua N, Fu X, Pan Y, Li B, Li X (2018). Metformin regulates atrial SK2 and SK3 expression through inhibiting the PKC/ERK signaling pathway in type 2 diabetic rats. *BMC Cardiovascular Disorders*.

[36] Liu C, Hua N, Fu X, Pan Y, Li B, Li X (2018). Metformin Regulates the Expression of SK2 and SK3 in the Atria of Rats with Type 2 Diabetes Mellitus through the NOX4/p38MAPK Signaling Pathway. *Journal of Cardiovascular Pharmacology*.

[37] Howarth FC, Qureshi MA, Jayaprakash P, Parekh K, Oz M, Dobrzynski H (2018). The Pattern of mRNA Expression is Changed in Sinoatrial Node from Goto-Kakizaki Type 2 Diabetic Rat Heart. *Journal of Diabetes Research*.

[38] Huang X, Zhong N, Zhang H, Ma A, Yuan Z, Guo N (2017). Reduced expression of HCN channels in the sinoatrial node of streptozotocin-induced diabetic rats. *Canadian Journal of Physiology and Pharmacology*.

[39] Hadova K, Kralova E, Doka G, Bies Pivackova L, Kmecova Z, Krenek P (2021). Isolated downregulation of HCN2 in ventricles of rats with streptozotocin-induced diabetic cardiomyopathy. *BMC Cardiovascular Disorders*.

[40] Sardu C, SANtamaria M, Rizzo MR, Barbieri M, di Marino M, Paolisso G (2016). Telemonitoring in heart failure patients treated by cardiac resynchronisation therapy with defibrillator (CRT-D): the TELECART Study. *International Journal of Clinical Practice*.

[41] Sardu C, SANtulli G, SANtamaria M, Barbieri M, Sacra C, Paolisso P (2017). Effects of Alpha Lipoic Acid on Multiple Cytokines and Biomarkers and Recurrence of Atrial Fibrillation within 1 Year of Catheter Ablation. *The American Journal of Cardiology*.

[42] Sardu C, SANtulli G, Guerra G, Trotta MC, SANtamaria M, Sacra C (2020). Modulation of SERCA in Patients with Persistent Atrial Fibrillation Treated by Epicardial Thoracoscopic Ablation: The CAMAF Study. *Journal of Clinical Medicine*.

